# Women health providers: materials on cures, remedies and sexuality in inquisitorial processes (15th–18th century)

**DOI:** 10.3389/fpsyg.2023.1178499

**Published:** 2023-07-10

**Authors:** Blanca Espina-Jerez, José Siles-González, M. Carmen Solano-Ruiz, Sagrario Gómez-Cantarino

**Affiliations:** ^1^Department of Nursing, University of Alicante, Alicante, Spain; ^2^ENDOCU Research Group (Nursing, Pain and Care), University of Castilla-La Mancha, Toledo, Spain; ^3^Faculty of Physiotherapy and Nursing, Toledo Campus, University of Castilla-La Mancha, Toledo, Spain

**Keywords:** history of nursing, gender, culture, witchcraft, traditional healers, health, sexuality

## Abstract

**Background:**

The first inquisitorial trials were against Muslims and Jews. Later, they focused on women, especially caregivers. Progressively, they were linked to witchcraft and sorcery because of their great care, generational and empirical knowledge. The historiography of health in the 15th–18th centuries still has important bibliographical and interpretative gaps in the care provided by women.

**Objective:**

To analyse the care provided by healers as health providers, accused by the Inquisition, justifying the importance of nursing in the diversity of community care in the 15th–18th centuries.

**Method:**

A scoping review was conducted following the Dialectical Structural Model of Care (DSMC). A database search was conducted for the period 2013–2022. Bibliographic and legislative resources were used. Cases and convictions from Castilla la Nueva were found in the National Historical Archive and the Diocesan Archive of Cuenca.

**Results:**

The concepts of healer, witch and sorceress envolved during the study period. They reflect and reveal the collective imaginary of the social structure. They had healing laboratories, practised psychological and sexual care. They used to accompany their therapeutic action with prayers and amulets. They shared their professional activity with their main denouncers, doctors, apothecaries and priests. They were usually women in socially vulnerable situations, who did not conform to social stereotypes.

**Conclusions:**

They were predecessors of today’s nursing, they overcame socio-cultural difficulties, although they were condemned for it. Healers did not manage to regulate their profession, but they acted as agents of health in a society that demanded them while participating in the “witch-hunt”.

## 1. Introduction

A1 culture is not only defined by its ability to affirm the principles that underpin it. There are also significant gestures or symptomatic traces, which sometimes go unnoticed because they are assumed without the need for any explanation, i.e. they are familiar. When they gain strength, they become true archetypes and crystallise in figurative representations, easily recognisable, without the need to question them (Siles, 2010).

This happened on a cultural level with women at the end of the late Middle Ages and during the Modern Age. Between the 14th and 18th centuries, the greatest persecution of women took place under the accusation of quackery, witchcraft and sorcery. They became structured in the collective imagination as a figure inspiring fear. The origin of this fear was none other than the very mystery of feminine nature, which posed a threat (Federici, 2021).

In this context, Pope Sixtus IV authorised the Spanish Catholic Monarchs to study and persecute heretics through the inquisitorial process. But what influence did the Tribunal of the Holy Office have on culture and social control in Spain from that time until its end in 1820? Because of its politic-religious status, it was supposed to restore the values of the Church to the moral and social order. However, once this objective was achieved, the Holy Office focused and harshly expanded its persecution on a large number of women, many of whom were dedicated to the care of the Church (Caro-Baroja, 2015). In this sense, there was a continuity between the persecution of women dedicated to care and the preceding persecution of heresy which, under the pretext of imposing religious orthodoxy, punished other forms of social subversion (Ginzburg, 1980).

After the first inquisitorial proceedings brought for reasons of faith against Muslims and Jews (15th–16th centuries), cases against women linked to physical and psychological care, although existing in the previous period, became even more prevalent in the 17th and 18th centuries (Pérez Ramírez, 1982; Sierra, 2005).

The social and cultural construction of the image that was generated of women dedicated to health and care during the XV-XVIII centuries defined a historical framework that must be seen from three angles: (1) *scientific*, and the consequent rejection of women being part of this world; (2) *religious*, where Christian tradition was an instrument of judgement; (3) *legal*, as an instrument of repression and punishment of dissident behaviour (Zamora Calvo and Ortiz, 2012).

Women of this period possessed knowledge of herbs, ointments and concoctions to treat different illnesses and different groups, children, women, men and the elderly, as well as different situations that altered the health-disease process (Ganso Pérez, 2017). Thus, in the face of their knowledge and their wide range of care, an irrational fear arose on the part of the three great powers of the time: medicine, the Church and the State.

The practices, herbs and remedies known to women were passed down through female, oral and generational channels, and thus their knowledge escaped male control. Their ability to intervene in people’s lives and health through *magical arts* could even cause harm to those who trusted in their knowledge or simply had a relationship with them. Thus, the people around them were even condemned (Green, 1989).

Women’s knowledge and practices were progressively marginalised, condemned and persecuted as they were identified with witchcraft. Thus, already in the 15th–18th centuries, witchcraft became clearly associated with the feminine, and a slow demonization of traditional beliefs began. The line separating folk healing practices from witchcraft, superstition and heresy was as thin as that between woman and witch (Federici, 2021).

The institutionalisation of medical knowledge from the twelfth century onwards separated women from the regulated practice of medicine and care, stigmatising their knowledge (Guerrero Navarrete, 2012). In addition to this, paganism and popular folklore followed two paths of adaptation to the new socio-political and religious situation. On the one hand, the Church appropriated certain pre-Christian traditions, such as the celebration of the bonfires of St. John. On the other hand, it added a series of preventive rites to festivities already impregnated with popular superstition. For example, to avoid the curses of witches at the beginning of Holy Week, the tradition of placing blessed bouquets on the doors and windows of homes on Palm Sunday was established (Pedrosa Bartolomé, 2012).

The Dominicans Heinrich Kramer and Jacob Sprenger, authors of the *Malleus maleficarum* or *Hammer of Witches*, published in Germany in 1486, the most appreciated synthesis of the classical treatises on magic, as well as of the mythology that had been developing since antiquity around witches. But its most important distinctive feature is that it is the work par excellence that triggered the witch-hunt. The authors fervently defended that witches were not the fruit of popular imagination; on the contrary, they were real beings, extremely evil, and had close links with the devil (Green, 1989; Guerrero Navarrete, 2012).

Given the enormous relevance and dissemination of this manual, it ended up becoming a legal instrument of the Royal Court of the Inquisition used to know, differentiate and judge a wide range of specifically female patients. It even went so far as to condemn doubly the midwife, in charge of conducting the birth and assisting the mother and the newborn, both for her condition as a woman and for her capacity for healing. Such a situation is perfectly described in the following excerpt from the *Hammer of* Witches:

”Let us consider women first of all; first, because this kind of perfidy is found in so frail a sex, more than in men. And our enquiry will be first of all general, as to the type of women who indulge in superstition and witchcraft; and thirdly specifically, with regard to midwives who surpass in malignity all others” (Kramer and Sprenger, 1847).

In order to understand the cultural and social situation of the history of women carers in the 15th–18th centuries, it is not enough to know the inquisitorial machinery, i.e. to glimpse the origins and foundation of the Inquisition, its nature and purpose, the territorial organisation, the number and quality of the trials, the conduct of commissaries, governors and other officials. It is necessary to examine the contextual, social and individual situation, to understand the concepts and attributions with which these women were filled with meaning, their qualities and character. It is therefore necessary to try to unravel through each trial, the specific facts for which they were tried, the testimonies, the crime attributed to them and the final outcome, acquittal or conviction.

Only in this way will it be possible to arrive at the intimate reason for many political and social events in the history of nursing during the Modern Age in Spain. It is a history of a dangerous panorama for the State in the time of the Catholic Monarchs, marked by the important social austerity of the Golden Age. This framework placed women in a vulnerable situation (single, widows, poor, etc.), having to survive with difficulty with domestic jobs or unregulated trades, under a way of life in which the relics of the ancestors of Christianity and ancestral paganism were intermingled.

Studies linking the Inquisition and health tend to focus on the figure of doctors, barbers and bleeders (Sarrión Mora, 2006; Arce and Damián, 2011; Martín Conty, 2015), few address the female perspective of care (Rojo Vega, 2012; Beltrán Muñoz, 2014) or tend to focus especially on the midwifery profession (Cabré i Pairet and Ortiz Gómez, 2001; García Martínez, 2012) and not so much on care with a broader vision. In fact, the persecution of women for reasons of quackery, witchcraft and sorcery is still one of the least studied phenomena in the history of Europe (Federici, 2021).

The history of nursing is a reflection of the history of women, as the female gender has been one of the main backbones of tradition and for the understanding of care in diverse populations. In the past, the right to access a minimum of literacy and much more higher education in different fields of study was reserved for only a privileged part of the population, generally men and sometimes wealthy women (Guerrero Navarrete). From this position, the situation of the female figures dedicated to care in the XV-XVIII centuries was and has been shaped.

For this reason, the present work acquires an important scientific relevance as it attempts to contribute to filling an existing gap in the diversity of the history of nursing, that of women as providers of health care and care in the Modern Age (15th–18th centuries). In order to do so, it is essential to resort to primary sources that can provide the closest possible version of the history of healers, witches and sorceresses, based on what is closest to the truth, their testimonies.

For this purpose, we will use the documents preserved in one of the most important communities and regions of the Inquisitorial period, Castilla la Nueva (Spain), which holds almost all the documents of the territory in their entirety. It should be noted that the jurisdiction of the Holy Office of Toledo was not limited to this province alone, but included the current dioceses of Toledo, Ciudad Real and Madrid-Alcalá (Spain). The Holy Office of Cuenca centralised the jurisdiction of Cuenca and Sigüenza (Spain). All these districts were grouped together in what is known as Castilla la Nueva.

The following section presents the methodology for carrying out the analysis according to the research objectives. The main objective was to analyse the care provided by healers as health providers, accused by the Inquisition, justifying the importance of nursing in the diversity of community care in the 15th–18th centuries. The secondary objectives were: (1) to differentiate the names by which the predecessors and health providers of today’s nursing were known during the 15th–18th centuries; (2) to determine the structure of care and the materials used for cures through the inventories made of the accused; (3) examine the physical and psychological care provided by women healers, taking into account social determinants, through the analysis of testimonies.

## 2. Materials and methods

### 2.1. Study design

In this article, a scoping review was carried out as a method to address the objective of the study, which was to analyse the care exercised by female healers accused by the Inquisition in the 15th–18th centuries. The scoping review is an optimal tool to determine the impact of a set of available publications and studies. In addition, it facilitates researchers to acquire a more focused view on the evaluation, synthesis and critique of primary documents (Munn et al., 2018). In addition, the JBI Critical Appraisal Checklist was used, specifically for systematic reviews and synthesis (Aromataris et al., 2015).

The Dialectical Structural Model of Care (DSMC) was used, which is suitable for studying the social and cultural history of care, fundamentally linked to the sexual and gender division of labour (Siles, 2010; Siles González and Solano Ruiz, 2016). For this research, it is of utmost importance taking into account the social and cultural aspects that interact in the assistance and care exercised by female healers in the 15th–18th centuries.

The DSMC makes it possible to analyse the care structures in order to subsequently establish relationships between them. The structures used were: (1) Functional Unit (FU), which represents the beliefs, knowledge and feelings of the people who live together and socialise within the same social structure, through which the social and cultural systems are constructed that determine the sexual and gender division of labour, which are so decisive in the health and care professions. In this case, it has to do with the concepts of *healer*, *witch* and *sorceress* that are articulated around the pseudo-professionalisation of care exercised by women. These reflect the beliefs, knowledge, norms, legal and professional limits as well as their evolution through the 15th–18th centuries; (2) Functional Framework (FF), which refers to the objects and space enabled to develop the activities of care, specifically, the materials of cures and uses known through the inventories of goods made to these women; (3) Functional Element (FE), integrates the social actors in charge of care as well as the care actions performed, in this research, the forms of physical, psychological and sexual care through the inquisitorial processes (Siles González, 2010; Espina-Jerez et al., 2022a,b).

**FIGURE 1:**
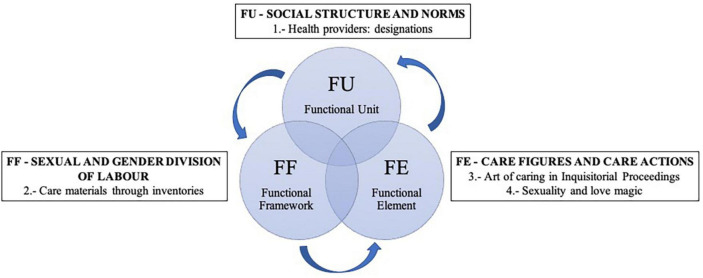
Theoretical dialectical structural model of care (DSMC): application of its structures. Source: authors’ own elaboration.

This research proposes four thematic blocks interrelated and analysed through the structures of the DSMC (Siles González and Solano Ruiz, 2016; Figure 1).

### 2.2. Search strategy

The review process began with an exploratory research question, aimed at systematically synthesising and critiquing existing knowledge (Colquhoun et al., 2014; Peters et al., 2015). In this case: “What was the impact of the three powers (Medicine, Church and State) on health care and health provider figures during the 15th–18th centuries?”

To answer this question, the DSMC was applied. The researchers agreed on the eligibility criteria, which had to contain information on the modern-day healer. The review included the different names under which the predecessors of today’s nurses and health care providers were known, as well as their evolution taking into account the social and cultural history during the 15th–18th centuries. In addition, we explored the cases of women whose property was inventoried in order to find out about the materials used to cure them, as well as other processes that reveal the physical and psychological care they provided in the social framework in which they lived.

The review included as primary sources inquisitorial processes from the period of study, 15th–18th centuries, dictionaries of the period and legislative material. Peer-reviewed articles, official dissertations, proceedings and reports were also included. Conference proceedings, proposals and editorials were excluded. The documents consulted were in English, Spanish and French.

We began with an initial search aimed at finding out the background and delimitation of the subject of the study. In this phase, various databases were consulted: (1) PubMed; (2) Cochrane; (3) Bibliographic database on health in Latin America (CUIDEN); (4) Scopus; (5) Web of Science; (6) SciELO.

For database browsing, a natural or free-text language was used, normalised and controlled with MeSH and DeCs descriptors. These were combined with Boolean operators (”and/and”, “or/or”, “not/not”). The results obtained and used, as well as the search equations and filters used to arrive at them, are summarised in Table 1.

**TABLE 1:** Thematic blocks related to search equations.

Database	Search strategy	Filters	Points extracted
Pubmed Cochrane Take care Scopus Web of Science SciELO	history of nursing AND witchcraft nursing AND witchcraft healer and nursing sorceress and nursing history of nursing AND gender AND legislation Modern Age and nursing healer AND inquisition witchcraft and Castile	Last 10 years Article English/Spanish	Health providers: designation Care materials through inventories Art of caring in Inquisitorial Proceeding Sexuality and love magic

Authors’ own elaboration.

After this initial search, we proceeded to consult manuals in physical and virtual format from different territories through the library service of the University of Castilla-La Mancha and the Toledo Public Library. In order to have up-to-date information, the search in database was limited to the last 10 years. However, due to the fact that this is a historical topic and perhaps not so prone to bibliographic updating, previous publications were consulted and selected due to their interest.

For the examination of dictionaries of the time as well as relevant legislative sources, the Miguel de Cervantes Virtual Library and Official State Bulletins respectively were consulted.

The inquisitorial cases and convictions of New Castile are essentially divided into two archives. The documents of the Courts of the Holy Office belonging to the archbishopric of Toledo are in the National Historical Archive (NHA), and those of the archbishopric of Cuenca are in the Diocesan Archive of Cuenca (DAC). For the consultation of documents held by the NHA, combined searches were carried out through the Spanish Archives Portal (PARES), entering the search terms and filtering by archive (NHA), Institutions of the monarchy and the Tribunal of the Inquisition of Toledo. On the other hand, the search in the DAC was carried out manually through the Catalogue published by Dimas Pérez (Pérez Ramírez, 1982).

The terms used in the PARES search after filtering the archive (NHA), Institutions of the monarchy and Tribunal of the Inquisition of Toledo, were: “healer”, “cure”, “cures”, “sorcery”. It was not necessary to filter the period of study as the “Tribunal of the Inquisition of Toledo” parameter itself already narrowed the search by date.

The search in the DAC followed a different procedure, as the catalogue is not digitised. A manual search was carried out, following the two existing records on the documents collected in the DAC, that of Sebastián Cirac Estopañán (1965) and that of Pérez Ramírez (1982), both contained in the same handbook (Pérez Ramírez, 1982). The chapters “Criminal Proceedings” and “Fourth series: Criminal Proceedings, II” were reviewed. In the search, the same terms were used as in the NHA.

**FIGURE 2:**
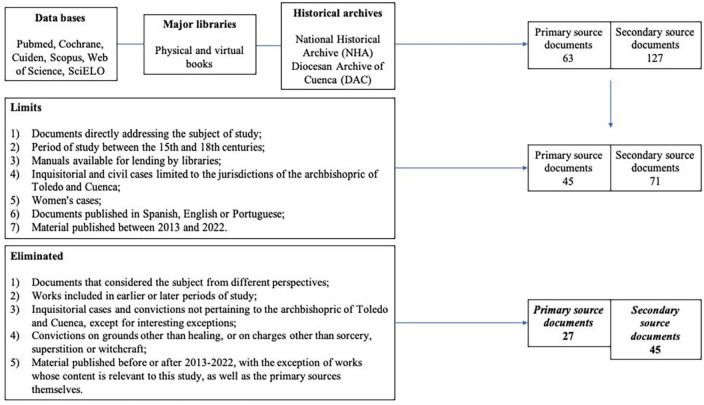
Filtering strategy. Source: authors’ own elaboration.

During the literature review and filtering process, a number of inclusion and exclusion criteria were applied (Figure 2). A total of 62 papers were found to meet the criteria.

### 2.3. Data analysis

The documentary analysis was conducted from a qualitative perspective, systematically following the objective of the study. The steps followed in the analysis were: (1) a thematic linkage; (2) a preliminary classification of the documents based on inclusion and exclusion criteria; (3) selection of relevant information; (4) interpretation and comparison of the results. The selected material was analysed from the point of view of the four thematic study blocks, each of them encompassed within the DSMC structures: (1) health providers: designations; (2) care materials through inventories; (3) art of caring in Inquisitorial Proceedings; (4) Sexuality and love magic. These blocks were contextualised in the Castilian population of the Modern Age (15th–18th centuries) in Castilla La Nueva (Spain).

To extract and summarise the data, the first and second authors conducted a general data extraction. The third author examined the findings in depth. The fourth author identified the four thematic blocks encompassed in the DSMC structures, from Functional Unit, Functional Framework and Functional Element. Discrepancies were resolved by consensus among the researchers. Thus, after working with all the material it was possible to answer the initial question of this study: “what impact did the three powers (Medicine, Church and State) have on care and health care providers in the 15th–18th centuries?”

## 3. Results

### 3.1. Health care providers: designations in the 15th–18th centuries. Functional unit

Depending on the place and time in history, women in care have been called by different names, so it is useful to focus on the terms and concepts before moving on to a more in-depth analysis.

According to the period on which the present study is focused, the 15th–18th centuries, the terms healer, witch and, sorceress the most commonly used to name what is known today as a *nurse*, will be broken down. In order to achieve an adequate approximation to the concepts according to their historical moment, the first dictionary of the Spanish language, published in 1611 by Sebastián Covarrubias Orozco, was used. (de Covarrubias Orozco, 1611) and the dictionary of the Castilian language of the Royal Spanish Academy (RSA) published in 1783 by Joaquín Ibarra (Royal Spanish Academy, 1783).

#### 3.1.1. Woman healer

It is noteworthy that the first dictionary does not contemplate the profession of *healer*, and only describes the term *cure*, which is defined as “to medicate a sick or injured person, because of the care that must be taken with him” (*to cure, which is* defined as “to medicate someone who is ill, or injured, by the care that must be taken with him”) (de Covarrubias Orozco, 1611).

On the other hand, the RSA does include the concept, except that it only applies it to the masculine gender. It determines that healer is “the one who introduces himself to give remedies and prescriptions without being an approved doctor” (Royal Spanish Academy, 1783). This meaning is quite interesting, as it does not reflect the fact that women in Castile who worked in the care professions in the 16th–18th centuries were left without regulated training and, consequently, at risk of legal and/or inquisitorial persecution (Espina-Jerez et al., 2022a). In the Modern Age, women were legally excluded from the caring sciences by being denied the opportunity to obtain adequate professional training (Clark, 1968).

#### 3.1.2 Witch and sorceress

**FIGURE 3:**
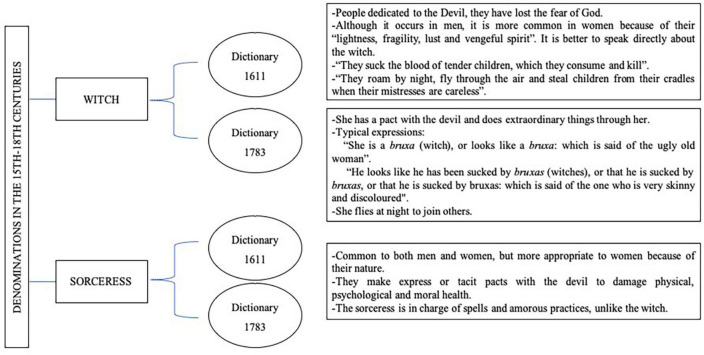
Witch and sorceress in the dictionaries of 1611 and 1783. Source: authors’ own elaboration based on de Covarrubias Orozco (1611) and Royal Spanish Academy (1783).

For the case of the *witch*, female or male, the Covarrubias dictionary (de Covarrubias Orozco, 1611) offers a very broad definition of the witch. Furthermore, to refer to their acts, he cites the *Malleus Maleficarum* and thus avoids going into further description. Years later, the dictionary of the RSA of 1783 (Royal Spanish Academy, 1783) completed and included some characteristics that the previous version did not include (Figure 3).

Regarding the term *sorceress*, it is only contemplated by the second dictionary analysed, that of 1783, however, its definition is implicit in both dictionaries with the word “enchant” (de Covarrubias Orozco, 1611; Royal Spanish Academy, 1783; Figure 3).

However, the distinction between witch and sorceress is not entirely clear. There is some controversy among current researchers. Lisón Tolosana (1992) differentiates between them according to their relationship with the occult and the perverse. He understands that the witch renounces her faith by making a pact with Satan, whom she worships, while the sorceress only invokes the demon to perform her spells and rituals. She also considers that both figures can coexist in one, and be both witch and sorceress, as in the case of Celestina (Lisón Tolosana, 1992), a Spanish comedy published in 1499 whose title corresponds to the name of the sorceress-witch. For Caro-Baroja (2015) the difference is established by the environment in which they carried out their activity: the witch in rural areas, victim of the “witch-hunts” of the XV-XVIII centuries, while the sorceress in urban areas (Caro-Baroja, 2015).

Undoubtedly, literary sources also socially represented the witch or sorceress, terms that will henceforth be used interchangeably in the text. In this respect, it is worth mentioning a fragment from one of Miguel de Cervantes’ Exemplary Novels (Rodríguez Cerdá, 2007) The Colloquy of the Dogs, which in one part of the work lists the multiple faculties and characteristics of a sorceress known as *the Camacha de Montilla*:

”You must know, son, that in this town lived the most famous sorceress that ever lived in the world, whom they called the Camacha of Montilla; she was so unique in her craft that the Eritos, the Circes, the Medeas, of whom I have heard that the histories are full, did not equal her. She froze the clouds when she wished, covering the face of the sun with them, and when she wished she made the most troubled sky serene; she brought men in an instant from distant lands, she wonderfully remedied the maidens who had neglected to keep their integrity, she covered widows so that they would be honest and dishonest, she unmarried the married women and married those she wanted to (.) She was famous for converting the women of Montilla, she was so unique in her profession that she was so unique that the Eritos, the Circes, the Medeas, whom I have heard of in stories full of them, did not equal her.) She was reputed to turn men into animals, and to have made use of a sexton for six years, in the form of an ass, really and truly, which I have never been able to find out how it is done, because what is said of those ancient magicians who turned men into beasts, those who know best say that she was not a magician, those who know best say that it was nothing more than that they, in their great beauty and with their flattery, attracted men in such a way that they loved them well, and subjected them in such a way, using them in everything they wanted, that they seemed like beasts” (Rodríguez Cerdá, 2007).

The premise of magic is that the world is alive, unpredictable and all things have something hidden in them that can be deciphered and manipulated according to one’s will (Wilson, 2000). Thus, the fragment shows how the sorceresses of the time can influence nature, prevent conception or save the moral honour of women, and intervene in the emotional situation of couples. Another accusation that is reflected is bestiality, that is, the transformation of men into beasts, a kind of male castration in a figurative or psychological sense.

In order to learn more about the characteristics of the figure of the Castilian witch or sorceress in the 15th–18th centuries, it is useful to refer to the character and the work of *La Celestina*, published in 1499 by De Rojas (2005). In most cases, these women fulfilled the stereotype of single or widowed women, sometimes of advanced age, experienced and who had to use various occupations to get by. Celestina had up to six professions, she was a sorceress, a procuress, a child physicist, a farmer, a perfumer and a master of making oils and virgins, that is, capable of restoring the hymen of women to make them believe that they were “virgins” (Montero Cartelle and Herrero Ingelmo, 2012). She was not a midwife, but a close friend of one of them, the mother of Pármeno. Her occupation was linked to gynaecology, obstetrics and the physical-emotional care of people without resources. In addition, the work alludes to the subject of abortion which, despite its express medical prohibition, was carried out given the frequent extra-marital relations in the 15th century, which were reprobated by Christian morality (Beltrán Muñoz, 2014).

### 3.2. Inventories of goods: the women healers’ laboratories. Functional framework

Through the inventories of goods made on the death of the women healers or when a process of faith was opened, it has been possible to verify that these women of the 15th–18th centuries used a diversity of objects and instruments in the exercise of their profession. The utensils and raw materials they used to prepare ointments, medicines, perfumes and everything they needed for cures were kept in the cupboards or corners of their kitchens. The majority of the processes analysed show that once the entire arsenal of these health care providers had been gathered, doctors and apothecaries were responsible for drawing up the list of goods and materials and interpreting their use (National Historical Archive, 1699, 1663, 1622).

The first laboratories found under the jurisdiction of Toledo and Cuenca date from the 17th century. However, it is pertinent to refer to the inventories of three of their predecessors found in the Provincial Historical Archive of Valladolid. The first of these is Beatriz de los Ríos (Provincial Historical Archive of Valladolid, 1584).whose inventory is dated 1584, and shows a list of objects that leads us to believe that she was quite precise in her measurements, in addition to her possible specialisation in ophthalmology (Table 2).

**TABLE 2:** Most relevant materials from the inventories to Beatriz de los Ríos and María Sánchez de la Rosa, and their usefulness.

Woman healer	Healing material	Utility
Beatriz de los Ríos (1584)	”A little iron weight with its scales made of sugar”.	Measurements
	”A glass vial with pink water”.	Eye wash
	”A glass jar with some powder in it”. “Two tiny little arches in which the curative powers are poured”.	Healing the eyes
	”A jar bathed in ointment from the *rijas*”.	Healing the eyes
	”A boot with medicines”	
	”Four pots of ointment	Healing the eyes
	”A pot and a jug, one with honey and the other with mead”.	
	”Plus a coloured pouch with a magnet stone”.	Mother sickness
	”Plus a chest in which he carries medicines”.	
María Sánchez de la Rosa (1693–1701)	Seed of *negrilla*	Facial blemishes
	Calamint or betony	Cicatrizant, astringent, treatment of migraines and neuralgia.
	Dried peppermint and sulphur flower	–
	A prescription paper	For various ailments
	A Talavera pot with black amber stones; crystalline stone; *Ymán* stone; *Ymán* stone.	–
	*Senna* leaves	Purgative medicine

Authors’ own elaboration from Provincial Historical Archive of Valladolid (1584) and National Historical Archive (1699).

The second case of interest is the inventory of María de Vega (1597), in which we find a whole pharmacological office at the service of the quackery. María was the owner of a water distillation workshop with nine stills, from which official medicinal waters were supplied to the local apothecaries. Among her list of assets, a water book and an accounting book were found (Provincial Historical Archive of Valladolid, 1597).

Something common in the inventories of these women is the number of beds, especially when they were widowed or lived alone. Possibly, the mattresses and other belongings were intended for the care of the sick, as in the case of Francisca Hernández. She ran a joint in which, when justice arrived to arrest her, they found a tavern, a noble couple making love, card players and, most interestingly, honest people who were secretly being anointed against syphilis. It was a place on the fringes of legality in different aspects, but as far as health was concerned, it was because the place where these treatments were applied was the Hospital de la Resurrección in Valladolid, where Cervantes’ *The Colloquy of the Dogs*, mentioned above, was located (Rojo Vega, 2012).

The first interesting laboratory found in the jurisdiction of Toledo and Cuenca was discovered in Madrid in 1622, in the house of Josefa Carranza. Among its curative materials of interest were a pot with resin and turpentine for women’s hips, fish (meconium), wheat, saffron, holy water, among other things (National Historical Archive, 1622).

**FIGURE 4:**
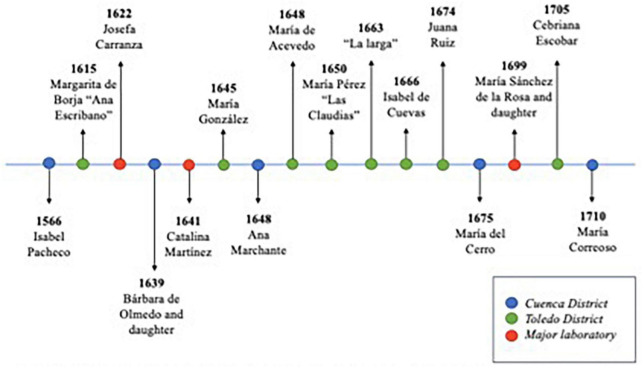
Timeline of women healers who were invented and found to have been used for cures. Source: Authors’ own elaboration from Diocesan Archive of Cuenca (1566, 1639, 1641, 1648, 1675, 1713) and National Historical Archive (1615, 1622, 1645, 1648, 1650, 1663, 1666, 1674, 1699, 1702a).

Many other laboratories were found in the area of Castilla la Nueva (Figure 4), however, the best equipped of all those discovered in the inquisitorial processes of the study area was that of María Sánchez de la Rosa. The apothecary Juan de Armuiña scrutinised a great variety of pots, pans, jars, papers with powders, ingredients and ointments (National Historical Archive, 1699; Table 2).

Maria also had human and animal bones, used as reliquaries, as was the custom at the time among sorceresses. It was also common to find religious holy cards. She kept them of Our Lord, Our Lady, St. Barbara and a *Lignum Crucis*, which the apothecary interpreted as relics of devotion (National Historical Archive, 1699).

### 3.3. Healers: evils, advice and prayers during the cure. Informers and sentences. A functional element

One of the ailments with which the population resorted to healers and/or sorceresses was the evil eye. This issue, which was already contemplated and had various remedies in the Andalusian medical treatises (Abu L-’Ala’ Zuhr (Avenzoar), 1994; Ibn Ḣabîb, 1992), was a cause of inquisitorial condemnation for many women, both for those capable of causing it and for those skilled in reversing it.

They were accused of being superstitious healers. In order to provide a closer look at this point, the cases of Isabel Rascalbo, Catalina Laso, Ana and Agustina, followed by the Inquisition Tribunal of Toledo, as well as that of Isabel Martínez followed by the Inquisition Tribunal of Cuenca, were analysed (Table 3).

**TABLE 3:** Women accused of being superstitious healers.

Name	Marital status	Source	Office	Date of process	Accusation	Sentence
Catalina Laso, “la Coracha”	–	Hita (Madrid-Alcalá, now Guadalajara)	Healer	1703–1704	Superstitious cures	–
Isabel Rascalbo, “la Valentina”	73 years old	Ciudad Real	Healer	1742	Superstitious healer, liar and panderer	Prisoner in the Royal Prison of CR
Isabel Martínez, “la dentera” (the toothache)	Single Lived together 32 years old	Tomelloso (Ciudad Real)	Healer	1726–1728 (1°) 1767 (2°)	Superstitious healer Sorceress Levi’s suspect	Prisoner in medium prisons and banishment (part 1)
Ana Díaz and Agustina Álvarez (mother and daughter)	–	Castañar de Ibor (Cáceres) Archbishopric of Toledo	Healers	1741–1745	Sorcery and superstitious cures	In abeyance

Authors’ own elaboration from National Historical Archive (1703, 1726, 1741, 1742).

#### 3.3.1. Catalina Laso: a case of conflict of interest

Misnamed “La Coracha”, she was accused of witchcraft and superstitious cures, which led to a trial of faith in the early 18th century (National Historical Archive, 1703).

When the Tribunal is informed of Catalina’s activities, an information letter is sent to the area commissioner, requesting further investigation of the elements of the statement with appropriate witnesses. The original letter states the following against Catalina:

”(.) there is a woman in the V^a^ de Hita who is misnamed la Coracha, who has the custom of sanctifying and cure animals, healing them with different things and the main one is with partridge feathers, and that she makes psalms and that to cure and to know the evil eye they bring her some jewels from the sick person and with them she knows the damage; and lastly, he does and says such nonsense that it has caused a great scandal in the town; and that the Apothecary and the priest of San Pedro and others that they could cite would testify at length about all this (.)”. (National Historical Archive, 1703) [1].

In this statement, in addition to the account of their practices, the complainants, both opponents, one in professional matters and the other in moral-religious matters, stand out. Finally, however, it is equally appropriate to highlight the conclusion of the commissioner who analysed and closed the case, who determined that the witnesses were not telling the truth.

#### 3.3.2. Isabel Rascalbo: plasters and prayers

Isabel Rascalbo in her cause called *”la R.”*, it is understood that by Rascalbo, declared in the prison of the same city, to have made different cures of gallic disease, mother’s disease and others, using chickpea water, mallows and other ingredients, without superstitious intentions and inclined to “do good” (National Historical Archive, 1742).

The Commissioner who handled the case noted in his report a paradox that was quite common for these women: on the one hand, they were well known, frequented and sought after by the population, while on the other hand, they were feared, mysterious and had a bad reputation:

”And it should be noted that the most said witnesses publicly testify and mention the bad work and reputation of the R. in the said city as a quack and shyster; and the Commissioner adds in his report that the same thing happens in all the places in the region, and that when the R. is mentioned, everyone is scandalised and fearful of her (.)” (National Historical Archive, 1742) [2].

In the case of Isabel Rascalvo, the testimony of seven witnesses is recorded, and as a sample, the testimony of two of them is shown (Table 4).

**TABLE 4:** Witnesses against Isabel Rascalvo.

Witnesses	Verbatim
*Informant 1* Francisca García. 22 years old. Single	”. she testifies that while serving in Ciudad Real, in the house of Phelipe Ruiz Casueros, and his mistress and wife, Mariana Sanchez, suffering some accident, she saw the R. frequenting the said house and staying alone with the sick woman for some time, but that the witness did not know what they were doing; that although on one occasion she would say that the R. made a fire in the room to make an ointment, she did not know what ingredients it was made of; she although said that the R. had a bad reputation in the said city of being a woman who knew how to make and unmake a spells”. [3].
*Informant 2* M^a^ Gregoria de la Cuadra. 25 years old. Married.	”. she testifies having heard in public and notorious that the R. was in common opinion to know how to cure and make prayers, make and unmake spell; that there will be about 4 or 5 years that the witness served in the house of Francisco Ruiz Carneros, neighbour of Ciudad Real, and his mistress Gerónima Sanchez wife of the referred one suffered severe accidents, the R. went to cure her staying some days in said house, that with effect it was experienced repeatedly that it made her well. However, the doctor said a thousand times that he could not achieve the protection of such evils; the witness could not (?) of the means by which the R. would be cured, but that on some occasions the R. took honey and put it on some tow, she applied it to the painful part, mixing certain powders on the honey that the informant did not know what they were”. [4.1]
	”… A few days later she saw the R. in her house treating a child of the evil eye and saying words that the informant did not understand. When she asked to the R. what prayer she was saying, the R. replied that it was the Gospel. The witness sourly reprimanding her for such nonsense and for treating her with gospels, told her that more credit should be given to her gospels than to the sacred ones, and that the witness, speaking with such blasphemy, left her”. [4.2]

Authors’ own elaboration from National Historical Archive (1742).

#### 3.3.3. Ana Díaz and Agustina Álvarez, mother and daughter, cure and cause the evil eye

Following the accusation, a trial was opened against them that lasted four years (1741–1745). Joseph Suárez de Herrera, declarant of the same town, informed the prosecutor of the Holy Office of the Archbishopric of Toledo about both of them in a letter. Joseph’s letter is certified before a notary, and signed by the latter, *Francisco Xavier de Lisnero*, which reveals Joseph’s own difficulty in doing so himself. He states that:

”… Ana and Agustina cure the evil eye and other illnesses, and give evil spirits in such a way that anyone who offends them in anything at the point of revenge, or in persons or properties, if they do not invite them to the vows, they prevent the consummation of the marriage and it is by going to them, and giving them something with certain prayers and crosses in a determined number that they cure” (National Historical Archive, 1741) [5.1].

”Likewise, some women complain that they lack milk and other natural things, and there are witnesses who can testify to this, and lastly, the whole town is full of fear because of these women, trying to give them what they ask for at the end, and not to give them what they ask for, because if they don’t do so, they will immediately take revenge: No corrections have remained for these women, neither from the priests nor from the religious” (National Historical Archive, 1741) [5.2].

The complainant then describes how his mother and daughter have affected him. He says that he started making soap three years ago and was very successful at it. However, one day, half a year ago, they had “some anger” and since then they have prevented him from making soap, “even though the materials had been put in the last perfection”, which is a great grievance for him as he then had difficulties in supporting his family. What is equally interesting is that “at the least diligence that they make, it is perfect”, which gives them the ability to make and unmake their curse at any time they wish (National Historical Archive, 1741).

#### 3.3.4. Isabel Martínez: the wax cake with prayers

The trial against Isabel Martínez lasted from 1726 to 1728. She was nicknamed “*la dentera*” (the toothache) for using teeth as an amulet. The cause of Isabel Martínez, whom the notary summarises as “*la Y*”, gained momentum when the bishop of Amiclan, with whom Isabel had secretly confessed, wrote to the Tribunal on 5 September 1726 with the confession she had made to him (Table 5).

**TABLE 5:** Witnesses against Isabel Martínez.

Witnesses	Verbatim
*Informant 1* Bishop of Amiclan	”(.) she was a widow who had an affair with a man, and that she wanted to get out of her bad state by marrying him and for this she asked him to interpose himself; And she also reported that she had cured many people who were cursed and had been taught by Cathalina Araque, deceased, and that for this she used a wax cake and a doll with wooden legs and arms, and that by placing the cake under a pebble, and the doll in her hand, she would say the prayer to San German and the bishop said that it was an invocation of the devil for other illicit means”. [6.1]
*Informant 2* Commissioner of Tomelloso (Ciudad Real) *Indirect implication* Local doctor Maria Rodriguez	”(.) giving an account of the diligences and proceedings that he made in the year 1719 by note that the doctor gave him against the Y, of which he did not give notice beforehand, as it seemed to this commissioner that it was all gossip and jealousy, but seeing that the Court has called the and what about them is that the doctor informed the commissioner what he heard M^a^ Rodríguez, that he and priest Ana Sacristán, having called the patient’s wife and that he and she offered to cure him because the said woman of the patient was not at home, and that he had preceded that he and, threatened Ana because took a hand fan from him, and that he entered between the two communication and unlawful” [6.2].

Authors’ own elaboration from National Historical Archive (1726).

Several witnesses acknowledged that Elizabeth cured different sick people. Once the doctor had indicated that there was no chance for the sick person, or even that he or she was bewitched, the relatives would turn to Isabel. According to the process, she managed to cure, usually with poultices and ointments *from the votica*, which were often accompanied by the wax doll and prayers recited in a low voice or in the absence of the relatives, with the intention of preserving her legacy and profession, as she acknowledged (National Historical Archive, 1726). Wax dolls for healing purposes were known as *votive offerings*. They were usually offered under divine promise by the families of sick people who requested their healing, and were then exhibited in temples and chapels as a reminder of the healing benefit (Cobo Delgado, 2016). In the case of Isabel, the witnesses also point out that she was able to estimate the number of days that the sick person had left to live, and once this had passed, the mourner would die (National Historical Archive, 1726).

Subsequent to these declarations, the Tomelloso police commissioner, Julio Alexandro, made a statement. He wrote to the Court justifying the fact that he had not previously denounced Isabel’s case, when the local doctor came to the commissioner to testify against her. He decided to do so when he heard María Rodríguez tell what had happened when Isabel was asked to go to a sick person’s house (Table 3).

Although most of the witnesses reported that Elizabeth provided welfare, and for some a positive outcome, she was judged to be suspected *of levi*, and was ordered to be imprisoned in medium prisons. As a prisoner, she asked to testify to *unburden her conscience* and told how she was initiated through Catherine into healing with the wax cake, the doll and the prayer to St. Germain:

”Glorious S. German, luck you cast for the sea, if good you cast it, good you took it out, run, and walk, galleys for the sea” (National Historical Archive, 1726) [7].

On this occasion, the prayer is collected because it is his own testimony, and it is noted that objectively it can be absolutely harmless.

### 3.4. Sexuality and love magic: types. A functional element

#### 3.4.1. Male impotence

One of the accusations common to these women, shared by Ana Díaz, Agustina Álvarez and Isabel Martínez, is related to what is known as love magic. The first two were said to be capable of preventing marriages from being consummated if they were not invited to weddings.

In the case of Isabel, one of the declarants accused her of having rendered the man with whom she was having relations impotent. The discourse is loaded with repeated events and magical justifications, so it is not surprising that the interviewer pointed out at the end that “the above-mentioned person gives signs of dishonesty in her declaration” (the aforementioned woman gives signs of dishonesty in her statement) (National Historical Archive, 1726) [8]. On the other hand, Isabel was living in an affair with Antonio Fernández, a misdemeanour condemned by the Inquisition, and for which Isabel herself confessed to the bishop, expressing her repentance and desire to change the situation. However, Antonio himself stated at his 1st and 2nd hearings that “(.) having taken leave of the Y (Isabel), he said that he had been driven mad because he could not rest without seeing her (.)” (National Historical Archive, 1726) which, applying logic, could be said to respond more to a natural desire than to a spell.

The belief that women could cause impotence in men was widespread. They were held to act by diminishing sexual desire or blocking their generative potency. They could even lead to what became known as *theft of the virile member* to hide them in large quantities in birds’ nests or boxes (Bueno Domínguez, 2012)until, under pressure, they were forced to return them to their owners (Federici, 2021). This issue was widely developed by European iconography of the time (Mattelaer, 2010).

Throughout the Middle and Modern Ages, impotence was considered a serious problem that went beyond medical knowledge. Andalusian midwives and physicians were familiar with many remedies to stimulate sexual potency in men and women, which are recorded in the recipe books of the period (Ibn Wâfid, 1980; Ibn Sa’îd, 1991). The knowledge of these women, coupled with the lack of medical training in this area, led to the search for sorceresses, who were held responsible for both inflicting and reversing it (Bueno Domínguez, 2012).

The case of Henry IV of Castile, nicknamed by some historians as *The Impotent*, who ended up accusing his wife Blanca of Navarre of being a witch when it was impossible for him to consummate the marriage, is well known. This situation degenerated into the granting of a marriage annulment to Blanca and the subsequent marriage to Juana of Portugal, from which his daughter Juana was born. Her paternity was questioned in the conflict of succession to the crown, as she was nicknamed the Beltraneja, given the rumours that she was conceived by Queen Juana and Beltrán de la Cueva (Ohara, 2004).

The sorceress created impotence by various means. It was even speculated that when a man was about to have a sexual encounter, an invisible body would come between him and the woman and prevent intercourse. It could also cool the man’s sexual arousal just at its peak, completely suppressing the erection of his member (Federici, 2021). The religious scholars Kramer and Sprenger threw out a somewhat logical, albeit biased, interpretation. Although the witches are responsible, rather than stealing the member, they make the man imagine that he does not have it, i.e., he is suggestible to the idea and possibly to previous failures in the sexual encounter. On the other hand, they alluded to the possibility that the demon, through the mediation of the sorceress, interfered with the senses and thus with the process of perception (Kramer and Sprenger, 1847).

Not only were women held responsible for male impotence, but female sexuality was made an object of fear. They could be accused of creating excessive erotic passion in men, so that it was easy for them to say that they had been bewitched or bewitched (Kors and Peters, 1972) as happened to Isabel Martinez when her ex-partner testified against her.

#### 3.4.2. Love filters: love me more or love me better

But love magic was not only about male impotence; love filters and love concoctions were also very popular. These were mainly used by women who wished to initiate, maintain or increase the affection of men. The practices and objects they used were diverse in nature: bodily elements, including hair and fluids, animals, plants, minerals, objects that could be tied together, such as ribbons or clothes, and prayers or incenses (Gómez Alonso, 2018).

Usually, something belonging to the applicant of the spell was used. In women, it was common to use armpit and pubic hair, as well as fingernails. Although it was even more common to use menstrual blood as an ingredient in the food or drink intended for the recipient of the spell (Gómez Alonso, 2018). The sorceresses Francisca Caballero (National Historical Archive, 1753), María Montoro and Catalina Ángel de Torres (National Historical Archive, 1789), as well as María Patiño (National Historical Archive, 1776) were accused by their own petitioners and prosecuted by the Toledo Inquisition for recommending the use of menstrual blood as a love spell, but they were not the only ones.

Among the foods popularly consecrated to love was the meat of the turtledove, a bird that medieval bestiaries considered a symbol of fidelity. One of the common forms of use was for the animal to come into contact with the woman’s genitals, prior to its preparation and offering to the beloved (Bueno Domínguez, 2012). Another main food or element of the Castilian sorceresses was the hen and its eggs, probably because they were easy to acquire. The aforementioned María Montoro and Catalina Ángel de Torres (National Historical Archive, 1789) used it in their spells.

However, women not only resorted to superstition to maintain or increase the love of a loved one towards them, but unfortunately they also did so to avoid mistreatment by their husbands. Isabel Fernández turned to María Romero so that, through Cebriana Escobar, she could obtain a remedy that would free her from the abuse and threats she suffered at the hands of her husband (National Historical Archive, 1702a,b). Cebriana, a 40-year-old widow, and María, friends and residents of Toledo, were forced to combine their work as seamstresses with witchcraft, given the low income they earned from their first job. Leonor Barzana (National Historical Archive, 1530) recommended to abused women that on Mondays, Wednesdays and Fridays they should pour their menstrual blood into the broth basin.

## 4. Discussion

As stated in the *Malleus Maleficarum* or Hammer of Witches (Kramer and Sprenger, 1847) although witchcraft also occurs in men, it is a woman’s own thing because of her inherent and innate physical and psychological characteristics. During the Modern Age, the debate on the “nature of the sexes”, which had already begun in the Middle Ages, was taken up again, leaving women stereotyped as weak in body and mind and biologically prone to the Devil. In this way, male control over women and the need for patriarchal order was justified (Green, 1989; Federici, 2021).

In the 15th–18th centuries, accusations of contraceptive practices, abortions, infanticide, sexual perversion and destruction of the generative potency of humans played a central role in the trials of women caregivers and in the imaginary of the witch (Federici, 2021). This can be seen in the descriptions in the *Malleus Maleficarum*, in the Spanish dictionaries of the time and in the trials collected in this study. The manual written by Kramer and Sprenger (1847) and the dictionaries of the historical period constitute the references that articulated the social and cultural systems of the 15th to 18th centuries, that is, the thoughts, beliefs, and feelings of the people who socialized with women providing care during this time, and who grouped themselves around the FU.

It is worth highlighting the evolution of the term witch in the second dictionary analysed. Although it directly defines the witch as feminine without explaining why, it refers to her attributions as “according to vulgar opinion” (Royal Spanish Academy, 1783). Something very interesting in this case is that it seems to indicate that the saying “be sucked of bruxas” does not actually occur, but rather refers to those who are thin or pale. This could occur in neonates or underweight infants, premature babies, babies with a disease or malnutrition, taking into account the conditions of poverty. In fact, in the 16th and 17th centuries, neonatal and infant mortality rates were very high. Thus, midwives and healers were accused of causing babies to die shortly after birth, to die suddenly or to be responsible for their illness despite the socio-sanitary conditions of the time (Murray, 1921; Federici, 2021; Espina-Jerez et al., 2022a). It also alludes to the popular belief that these women could fly and gather in witches’ covens, which some authors (Murray, 1921; Cohn, 1980; Ginzburg, 2003, 1991) indicate that it was nothing more indicate that this was no more than a pre-Christian practice of fertility worship.

On the other hand, the boundaries and conceptual abstraction between the figures of witch and sorceress in Spain are blurred in their own time, as can be seen in dictionaries, and in the present day, as anthropologists and historians (Lisón Tolosana, 1992; Caro-Baroja, 2015) are unable to find agreement on the terms. In part, this may be due to the fact that the Inquisition itself did not distinguish between them in its trials and condemnations (Zamora Calvo and Ortiz, 2012), as can be seen in the trials collected in this study.

Following Ginzburg’s (1980) theory, it is possible to observe the presence of institutionalized and official care, as well as non-institutionalized care rooted in popular culture. This common circumstance in the study of nursing history is articulated in the interrelationship between the FU and the FF, resulting in the final FE that is analysed. The diverse population of New Castile in the 15th–18th centuries resorted to natural and supernatural means to improve, maintain or restore their physical and emotional health. When the first means failed, or were physically or economically inaccessible, they resorted to a supernatural explanation and cure. This happened in the case of Isabel Martínez, alias “la Y”, in whose trial witnesses acknowledge that she cured several sick people once the doctor had not found a scientific solution.

Something that is sometimes left to be seen in the trials is the veracity of the witnesses, as happened in the case of Catalina Laso. In this process, the commissioner ends by saying that the witnesses “are very untruthful” (National Historical Archive, 1703). In many cases, the denouncers are people with a clear conflict of professional interests. Catalina Laso was denounced by the apothecary, the scientific power, and the priest of San Pedro, her village, who held ecclesiastical power. The same happened to Isabel Martínez, who was initially denounced by the local doctor, to whom the commissioner paid no attention, and later by the Bishop of Amiclan, making use of the allegedly secret confession that Isabel Martínez had had with him.

For Federici (2021) the persecution of women was an attack on the power they had gained through the ability to heal, sexuality and control reproduction. In Essex (England) and other rural areas of England, peasant and poor women were accused by wealthy and prestigious members of the community, thus individuals who were part of the local power structures (Macfarlane, 1970). Similarly, in the case of the women in this study who were accused of quackery and sorcery, the power figures of the time, doctors, apothecaries and priests, prevailed as accusers. This illustrates the sexual and gender division of labour in an interrelationship between the three structures of the DSMC.

In the testimonies against Isabel Rascalbo and Isabel Martínez, the witnesses acknowledge that they do not know the care practices, the ingredients of their medicines and the prayers that sometimes accompanied the cure. However, such a situation is not surprising if one takes into account that for them it was an unprofessionalised profession and, therefore, the transmission of their knowledge was purely oral (Zamora Calvo and Ortiz, 2012; Espina-Jerez et al., 2022a), and on many occasions, passing from mother to daughter, as in several of the processes discussed here. On the other hand, the durability of the care provided by a declarant in the proceedings against Isabel Rascalbo and Isabel Martínez is striking. The latter stayed at the home of the mourner for several days until the respective patients got better.

The ingredients and healing materials were accessed through the inventories of goods taken from these women. In many cases, the objects take on a superstitious tinge, but in others, the scientific rigour of the ingredients used and the measuring and preparation instruments can be observed.

In many cases, the prayers used during the cure were the same as the sacred ones. However, it is noted that this could be one of the grounds for condemnation when they were used for healing outside the ecclesiastical sphere. For example, the prayer to St. Germain that “the Y” performed while treated was interpreted by the church as an invocation to the devil, rather than a cure by Christian mediation as traditionally done.

The marital status of these women was a social determinant of gender. Being a widow and living in an affair was considered a sexual misdemeanour (Sierra, 2005) which like Isabel Martínez, could have consequences for them. Since neither the training nor the trade was regulated (Espina-Jerez et al., 2022a) and they were poor women, they did not have the resources to cope otherwise and defend themselves.

Healers, witches and sorceresses were endowed with great powers and abilities to cause, justify and undo any evil that afflicted the population (López-Muñoz et al., 2008; Federici, 2021). It was said that Isabel Rascalbo knew how to cast and undo spells (National Historical Archive, 1742). Isabel Martínez was said to be able to guess the day on which a person would die. Ana Díaz and Agustina Álvarez were blamed for the fact that some women lacked milk and “other natural things”. A declarant whose trade was soap making was prevented by his mother and daughter from continuing his work until he forced them to give him back his soap-making skills (National Historical Archive, 1741).

Likewise, the intervention of these women prevented the consummation of the marriage and produced male impotence (Gómez Alonso, 2018), another care action of a psychological nature (FE). However, the main consultants of love magic in Castile were women, who asked to maintain or increase the love of their husbands, or to avoid mistreatment. It is most likely that few or none of the above remedies had the expected direct effect, which is why in most cases the sorceresses were denounced. What did occur, however, was a framework and a secret environment for women to share and support each other’s emotional suffering, whether it was due to heartbreak or abuse.

The DSMC theory (Siles González and Solano Ruiz, 2016) has allowed the classification of the various phenomena surrounding the figures of care during the 15th–18th centuries, as well as the care provided by them. For the study of social and cultural history, it is necessary to clarify the social processes that are articulated around what is regulated at the macro level (FU). In this case, explicit and implicit norms regulate the way society thinks and acts during the 15th–18th centuries. It is important to take into account where care actions take place and whether or not there is a sexual and gender division of labour (FF), which is so present in nursing history during this period. Finally, the actors and their health actions (FE) should be considered, trying to give a place to the invisible women healthcare providers in history, in this case, midwives and healers, who combined physical care with a folk framework, under the demands of a society that requested their services.

In terms of primary sources, property inventories and testimonies are the only way of gaining direct access to these women in the first person. However, in the case of testimonies, as Ginzburg (1991) pointed out, the victims’ point of view is somewhat biased if we take into account that all that remains of their voices are the confessions written by the inquisitors, in many cases obtained under threat and/or torture.

## 5. Conclusion

The boundaries between one type of healing and another were never defined, but often coexisted, sometimes harmoniously, sometimes in conflict. Similarly, physical and emotional healing could go hand in hand, for sometimes the emotional spell had caused a physical ailment, or vice versa.

The health providers were immersed in the social nucleus in which they assisted. Some were highly trained for the job, while others, even if they were, combined their technical performance with a more popular and empirical knowledge typical of the rural world. The latter usually introduced materials, rituals or prayers that were considered superstitious by the Church. On most occasions, the elements were similar to those used in the ecclesiastical sphere, such as votive offerings, offered as a promise to obtain divine healing. Common saints were usually prayed to in prayers, and yet the very act of praying to them outside the framework of the Church was judged as an invocation of the devil. This double standard reveals the competition between the powers of the time (State, Religion and Medicine) for social dominance, displacing and condemning the activities of healers.

The persecution of women was an attack on the power they had gained in healing, sexuality and control of reproduction. People who turned to a healer or sorceress sought to keep away from harm and to achieve good, which consisted of well-being, health, life-support, fertility and so on. Even when they were not expert healers or sorceresses, they were called upon to heal their neighbours, give them amulets or potions for love, predict their future, and so on.

The use of their wisdom undermined the power of other regulated health figures and those belonging to church and state. The prosecution of women caregivers was directed at a wide variety of women’s practices. However, it should be noted that these women were actually persecuted for being the ones who carried out these practices, as sorceresses, healers, witches, soothsayers, etc.

If we look at the social determinants, we see that these women, in addition to generally living in rural areas, tended to be single or widowed. They lived in a situation of poverty, which meant that they had to work in various trades in order to survive. This combination of situations placed women, and in particular those dedicated to caregiving, in a double situation of social and inquisitorial vulnerability.

The care exercised by women has not always been studied from a perspective free of prejudices surrounding the popular. For this reason, it was of vital importance to analyse and discuss the primary sources, taking into account social structures, gender as a structural determinant, religious, economic and symbolic factors and their mentalities, under the dialectic between the official health culture and the popular culture.

The predecessors of today’s nurses overcame difficulties and socio-cultural barriers, but they were also condemned for it. The healers of the time did not manage to regulate their situation, but they acted as health agents in a society that demanded them and at the same time condemned them for the slightest error, without any kind of cover or support, on the contrary, with an inquisitorial network of “witch-hunting”.

The situation of these women reflects the fact that social change cannot only go in hand with professionals and recipients, but requires political transformations that cut across society. History takes us on a journey between before and after, through the legacy received from the anonymous women caregivers in today’s recognised health professions. However, there is still a long way to go in a profession in which the majority are women, and in which we still see various phenomena of inequality and inequity within and between professions.

## Author contributions

BE-J, JS-G, MS-R, and SG-C: conceptualization. BE-J and MS-R: methodology. SG-C and BE-J: formal analysis. BE-J and SG-C: writing – original draft preparation. BE-J, JS-G, and MS-R: writing – review and editing. MS-R and SG-C: supervision. All authors have read and agreed to the published version of the manuscript.
