# Supplementary material for: Women health providers: materials on cures, remedies and sexuality in inquisitorial processes (15th–18th century)
Source: Front Psychol. 2023 Jul 10;14:1178499. doi: 10.3389/fpsyg.2023.1178499 (PMC10364442; doi:10.3389/fpsyg.2023.1178499)
Supplement: Supplementary file 1 [file Data_Sheet_1.DOCX]

Supplementary Material

Women health providers: materials on cures, remedies and sexuality in inquisitorial processes (15th-18th century)

Blanca Espina-Jerez^*^, José Siles-González, M. Carmen Solano-Ruiz, Sagrario Gómez-Cantarino

*** Correspondence:** Blanca Espina-Jerez: bej1@alu.ua.es

# Supplementary Data

The original statements are included in the Spanish of the period under study, which is of interest because of its distinctive characteristics. In the text of the main document they are numbered in brackets.

[1] “(…) ay una muger en la Vª de Hita que por mal nombre la llaman la Coracha la qual tiene costumbre santiguar y curar animales, saumandoles con diferentes cosas y la principal es con plumas de perdiz, y que haze ensalmos y que para curar y conocer de mal de ojo le traían algunas alajas de la persona enferma y con ellas conoce el daño; y por último hace y dice tales disparates queadado mucho escandalo en la villa; y que de todo esto depondrían por extenso el Boticario y el cura de San Pedro y otros que estos citaran (…)” (Archivo Histórico Nacional, 1703).

[2] “Y es de notar que los mas dichos testigos deponen de publico y nombran la mala labor y fama en que está la R. en dicha Ciudad de curandera y echicera; y añadiendo el Comisario en su Informe que lo mismo sucede en todos los lugares de la comarca, y que nombrando a la R. todos se escandalizan y la temen (…)” (Archivo Histórico Nacional, 1742)*.*

[3] “… depone que estando sirviendo en Ciudad Real, en casa de Phelipe Ruiz Casueros, y padeciendo su ama y muger del referido, Mariana Sanchez algun accidente, vio frecuentar a la R en dicha casa y quedarse a solas con la enferma por algún tiempo, pero que no sabia la testigo lo que hacían; que aunque en una ocasion la pasaría que la R. hizo lumbre en el quarto para echar un samuerzo, no sabía de que ingredientes se componía; bien que la R. estaba en mala reputación en la dicha Ciudad de ser muger que sabia hacer y deshacer echizos” (Archivo Histórico Nacional, 1742).

[4.1] “… depone haber oído en público y notorio que la R. estaba en opinión común de saber curar y hacer ensalmos, hazer y deshacer echizos; que habrá como 4 o 5 a. que sirbiendo la testigo en casa de Francisco Ruiz Carneros, Exmo vecino de Ciudad Real, y padeciendo su ama Gerónima Sanchez muger del referido grabes accidentes, fue la R. a curarla quedándose algunos días en dicha casa, que con efecto se experimento repetidas veces que la ponía buena. Sin embargo, de que el medico dixo mil veces no podía alcanzar de que la protejan tales males; la que testigo no podrá (¿?) de los medios de que habrá la R. curar, si que en algunas ocasiones cogia la R. miel y puestas sobre unas estopas, las aplicaba en la parte dolorida, mezclando sobre la miel ciertos polvos que la deponente no sabia de que fuesen” (Archivo Histórico Nacional, 1742).

[4.2] “(…) fue pasados pocos días que vio estar la R. en su casa curando a un niño de mal de ojo y que hacia curas y decia palabras que no entendio el deponente, que preguntada la R. que esa oración que decia, respondio que era Evangelio y que reprendiendola agriamente el testigo de tales disparates y de que ella curase de evangelios, le dixo que mas crédito se debía dar a sus evangelios que a los sagrados, y que hablando el testigo con tal blasfemia la dejo” (Archivo Histórico Nacional, 1742).

[5.1] “(…) Ana y Agustina que curan de mal de ojo, y otras enfermedades, y dan malefizios de suerte que qualquiera persona que las ofende en algo al punto se vengan, ô en personas, ô aciendas, si no las convidan â las vodas, impiden el consumar el matrimonio y es acudiendo, y dandolas alguna cosa con ciertas oraciones, y cruces en numero determinado curan” (Archivo Histórico Nacional, 1741).

[5.2] “Asi mismo se quexan algunas mugeres que les falta la leche, y otras cosas naturales, lo que ay testigos que pueden declarar sobre esto, y por ultimo todo este pueblo esta lleno de temores por causa de estas mugeres procurando cada final darles quanto piden, y no despuntarlas, porque de no hazerlo assi, al punto se vengan: No han restado correcziones para estas mugeres, ni de Curas ni de Religiosos” (Archivo Histórico Nacional, 1741).

[6.1] “(…) fue viuda amancebada con un hombre, y que queria salir del mal estado casandose con el y para esto pedia se interpusiese; Y tambien comunico que avia curado a muchos maleficiados y la avia enseñado Cathalina Araque, difuncta, y que para ello se valia de una torta de cera y un muñeco de piernas y brazos de palo, y que puesta la torta bajo de un guijarro, y el muñeco en la mano, decia la oración a San German y el obispo allo que era una ymbocación del demonio para otros medios ylícitos” (Archivo Histórico Nacional, 1726).

[6.2] “(…) dando quenta de las diligencias y autos que hizo en el año 1719 por nota que le dio el médico contra la Y, de que no dio antes aviso pareciendole a este comisario que todo era chisme y celos, pero viendo que el Tribunal a llamado a la Y, les remite: Y lo que de ellas consta es que el médico delató ante el comisario lo que oyó a Mª Rodríguez, de que la Y curaba a Ana Sacristán, habiendo sido llamada de la muger del enfermo y que la Y avia ofrecido curarlo con que la dicha muger del enfermo no estuviese en casa, y que avia precedido que la Y, avia amenazado a Ana porque la avia quitado un avanico, y que avia entre los dos comunicación ylicita” (Archivo Histórico Nacional, 1726)..

[7] “Glorioso S. German, suerte echastes para la mar, si buena la echaste, buena la sacaste, correr, y andar, galericas para la mar” (Archivo Histórico Nacional, 1726).

[8] “(…) aviendose apartado la Y (Isabel), decia estava echizado porque no sosegaba no viéndola (…)” (Archivo Histórico Nacional, 1726).
